# Secondary Bioactive Metabolites from Foods of Plant Origin as *Theravention* Agents against Neurodegenerative Disorders

**DOI:** 10.3390/foods13142289

**Published:** 2024-07-20

**Authors:** Telma Marisa Gomes, Patrícia Sousa, Catarina Campos, Rosa Perestrelo, José S. Câmara

**Affiliations:** 1 CQM—Centro de Química da Madeira, NPRG, Universidade da Madeira, Campus da Penteada, 9020-105 Funchal, Portugal; telma.gomes@staff.uma.pt (T.M.G.); patricia.sousa@staff.uma.pt (P.S.); catarina.campos@staff.uma.pt (C.C.); rmp@staff.uma.pt (R.P.); 2 Departamento de Química, Faculdade de Ciências Exatas e Engenharia, Universidade da Madeira, Campus da Penteada, 9020-105 Funchal, Portugal

**Keywords:** phytochemicals, secondary bioactive metabolites, neurodegenerative effects, Alzheimer’s disease, Parkinson’s disease, therapeutic and preventive strategies

## Abstract

Neurodegenerative disorders (NDDs) such as Alzheimer’s (AD) and Parkinson’s (PD) are on the rise, robbing people of their memories and independence. While risk factors such as age and genetics play an important role, exciting studies suggest that a diet rich in foods from plant origin may offer a line of defense. These kinds of foods, namely fruits and vegetables, are packed with a plethora of powerful bioactive secondary metabolites (SBMs), including terpenoids, polyphenols, glucosinolates, phytosterols and capsaicinoids, which exhibit a wide range of biological activities including antioxidant, antidiabetic, antihypertensive, anti-Alzheimer’s, antiproliferative, and antimicrobial properties, associated with preventive effects in the development of chronic diseases mediated by oxidative stress such as type 2 diabetes mellitus, respiratory diseases, cancer, cardiovascular diseases, and NDDs. This review explores the potential of SBMs as *theravention* agents (metabolites with therapeutic and preventive action) against NDDs. By understanding the science behind plant-based prevention, we may be able to develop new strategies to promote brain health and prevent the rise in NDDs. The proposed review stands out by emphasizing the integration of multiple SBMs in plant-based foods and their potential in preventing NDDs. Previous research has often focused on individual compounds or specific foods, but this review aims to present a comprehensive fingerprint of how a diet rich in various SBMs can synergistically contribute to brain health. The risk factors related to NDD development and the diagnostic process, in addition to some examples of food-related products and medicinal plants that significantly reduce the inhibition of acetylcholinesterase (AChE), butyrylcholinesterase (BChE), and β-site amyloid precursor protein (APP) cleaving enzyme 1 (BACE1), are highlighted.

## 1. Introduction

**Figure 1:**
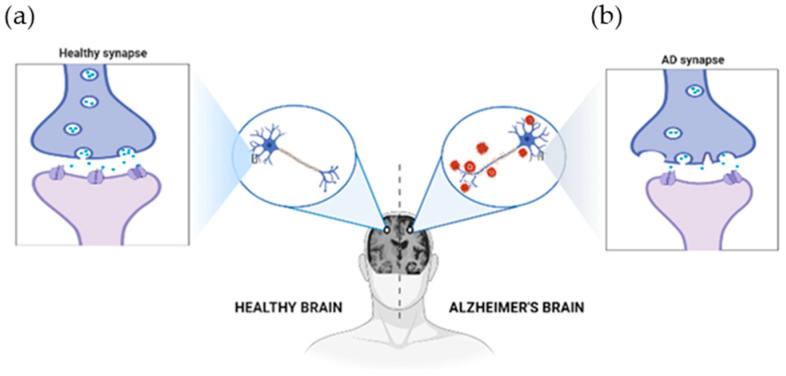
The physiological structure of the brain and neurons in (**a**) a healthy brain and (**b**) an Alzheimer’s disease brain. The healthy brain is full of neurons, each with healthy synapses. In Alzheimer’s, these connections are lost, and harmful clumps (plaques and tangles) build-up. This disrupts the brain’s work, leading to Alzheimer’s symptoms. Created with BioRender.com.

Neurodegenerative disorders (NDDs), characterized by progressive degeneration of the structure and function of the nervous system, pose significant health challenges worldwide. Among these, Alzheimer’s disease (AD) and Parkinson’s disease (PD) are the most prevalent, severely impacting cognitive and motor functions, respectively. AD is a slowly progressive NDD, characterized by dementia. Is estimated that, worldwide, over 55 million people suffer from dementia, with AD being the most common form (60–70% of cases) [1]. This pathology has a great impact, not only on the patient’s life and his family, but also on the financial level of society, which is estimated to cost USD 1 trillion annually at a global level [2]. Dementia is characterized as a cognitive impairment that affects the independence and daily life of patients. The impairment of cognitive functions can be a result of a variety of multiple neurodegenerative and cerebrovascular pathologies such as infections or abnormalities in the pulmonary and circulatory systems, due to a reduction of the oxygen supply in the brain, nutritional deficiency, vitamin B12 deficiency, and/or tumors, particularly in older patients [2,3]. AD usually results in a progressive loss of memory and cognitive functions that can include deficiency of language and visuospatial abilities, and that are often accompanied by behavioral disorders such as apathy, aggressiveness, and depression. AD relentlessly attacks the brain through a cascade of protein misfolding. Hyperphosphorylated tau and beta-amyloid aggregate into tangles and plaques, respectively, disrupting neuronal structure and function. Microglial activation represents a futile attempt to clear this debris. Meanwhile, synapses are degraded, neurons die, and essential neurotransmitters such as acetylcholine are depleted. Disturbed calcium homeostasis and oxidative stress further exacerbate neuronal death. These ‘positive’ (accumulation) and ‘negative’ (loss/dysfunction) lesions cripple brain networks, leading to the hallmark cognitive decline of AD, Figure 1 [2].

PD is the second most frequent NDD, affecting around 10 million people worldwide, 1% of those between the ages of 60 and 64 years old to up to 4% of those aged 85 years and older [4]. This NDD has a wide range of clinical characteristics, including motor (e.g., tremors, bradykinesia, stiffness, postural instability) and nonmotor (e.g., depression, cognitive impairment, sleep disturbances) symptoms, and can lead to diminished quality of life, disability, or even mortality in the elderly. This NDD is defined by the selective death of dopaminergic neurons in the substantia nigra pars compacta, although the precise cause is unknown. The data suggest that cerebral and peripheral inflammation play important roles in the pathologic characteristics and symptoms of PD [5,6,7]. In response to these debilitating conditions, various therapeutic and preventive strategies (theravention strategies) have been developed to manage symptoms, slow disease progression, and improve the quality of life for affected individuals. An emerging area of interest in this field is the use of secondary bioactive metabolites (SBMs), particularly polyphenols, anthocyanins, terpenoids, alkaloids, sulforaphane, glucosinolates, vitamins, polyunsaturated fatty acids (PUFA), and fibers, which are found in fruits, vegetables, grains, and other plant-based foods, in the prevention of NDDs. These SBMs have shown promise in neuroprotection with minimal toxicity due to their antioxidant, anti-inflammatory, and anti-apoptotic properties, allowing for neuronal growth factor accessibility, neurotransmitter homeostasis, and reducing neurotoxicity by preventing cerebral inflammation and oxidative stress [8,9,10]. For these properties, SBMs are being increasingly studied for their potential to mitigate the effects of NDDs and offer a complementary approach to conventional therapies. Research continues to explore the efficacy and mechanisms of these SBMs, aiming to harness their full potential in the fight against NDDs.

This review covers the risk factors associated with NDDs, the current diagnosis, and the potential of SBMs from plant-based foods as *theravention* agents for NDDs. In addition, the health-promoting effects of SBMs found in plant-based foods and herbs against NDDs are also discussed. This section not only identifies promising SBMs but also elaborates on their mechanisms of action and bioavailability. It explores how these SBMs interact at the molecular and cellular levels to exert their protective or therapeutic effects on neural tissue.

### 1.1. Risk Factors Associated with Neurodegenerative Disorders

**Figure 2:**
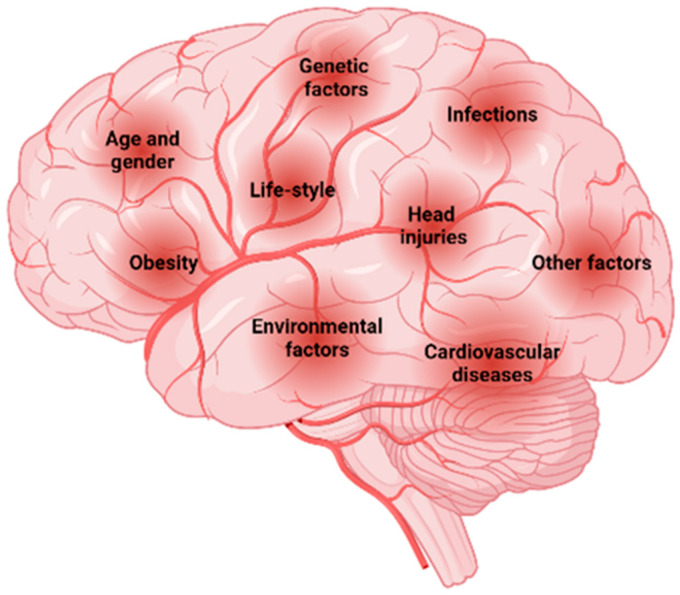
Risk factors associated with NDDs. Created with BioRender.com.

The risk factors associated with NDDs (Figure 2), including AD and PD, integrates a wide variety of components that can have major impacts on the onset and progression of these disorders [11]. These include genetics, age advancement, lifestyle, comorbidities, traumatic brain injury, and environmental factors such as exposure to smoking, pesticides, and other toxins [12]. In addition, some dietary factors may have a potential preventive function.

#### 1.1.1. Aging

Psychological disorders are associated with a large increase in morbidity in the elderly [13]. Consequently, aging, which is often associated with an increase in systemic inflammation, is considered a significant risk factor for cognitive decline. Most cases of these diseases, such as AD and PD, develop after the age of 65, indicating a late onset. The brain loses weight, volume, and synapses with age. Aging is also associated with glucose hypometabolism, lipid dysregulation, mitochondrial dysfunction, depression, and cognitive impairment [14,15,16].

#### 1.1.2. Genetic Factors

The development and progression of AD and PD are influenced by a complex combination of genetic and environmental factors. In AD, the ԑ4 allele of the APOE gene is a major genetic risk factor inherited in an autosomal dominant pattern. Other key genes implicated in AD include presenilin-1, presenilin-2, and the triggering receptor expressed on myeloid cells 2, which are involved in the production and processing of β-amyloid protein, leading to neurodegeneration and cognitive decline [17,18]. Similarly, PD has a diverse genetic basis, with both familial and sporadic forms. Familial PD is associated with mutations in genes such as alpha-synuclein, leucine-rich repeat kinase 2, glucocerebrosidase, and PARKIN (e.g., PARK2, PARK6, PARK7). Common genetic variants also contribute to the hereditary risk for sporadic PD [17]. These genetic factors interact with environmental influences to shape the onset and progression of these NDDs [18,19,20].

#### 1.1.3. Environmental Factors

Environmental factors, such as air pollution, heavy metals, and pesticides, can significantly influence the development and progression of NDDs. Air pollution, consisting of various pollutants, can lead to oxidative stress, neuroinflammation, and neurodegeneration, contributing to the conformational changes in disease-related proteins such as Aβ, tau, and α-synuclein [14,17]. Minerals, including zinc, manganese, iron, and copper, also play a pivotal role as essential enzyme cofactors in the normal functioning of the central nervous system (CNS), supporting key processes like neurotransmission, cellular metabolism, and antioxidant defense. Dysregulation of these critical elements can lead to protein aggregation, oxidative stress, and mitochondrial stress, all of which have been strongly linked to the development and progression of NDDs including AD and PD [21]. Pesticides and herbicides, particularly rotenone, paraquat, and maneb, are the most recent and controversial neurotoxins used to model PD [22]. Additionally, certain viral infections such as herpes simplex virus and bacterial infections (e.g., *Treponema pallidum*, *Chlamydia pneumoniae*) may be associated with an increased risk of developing NDDs [14]. These environmental factors can permeate the blood-brain barrier, affecting central nervous system cells and disrupting brain function, ultimately increasing the risk of NDDs through various molecular pathways [18].

#### 1.1.4. Dietary Factors

High-heat processing methods like frying, grilling, and roasting not only degrade valuable micronutrients in food, but also contribute to the formation of advanced glycation end products (AGEs). These harmful byproducts can also be formed during some high-temperature drying techniques. Conversely, methods like freeze-drying minimize heat exposure and limit AGE formation. Consuming foods high in AGEs, formed during processing or high-temperature cooking, can contribute to oxidative stress and inflammation. These damaging effects, caused by altering the structure and function of cell surface receptors, have been linked to cognitive decline and the progression of AD and PD [23]. The accumulation of AGEs can cause neuronal damage and impair cellular function, exacerbating the symptoms and severity of NDDs. Therefore, reducing AGE production through dietary and lifestyle changes may help reduce the influence of these harmful chemicals on the course of AD and PD [14,24]. Healthy dietary components such as polyphenols (e.g., curcumin, resveratrol), vitamins (e.g., vitamin B12, vitamin D), terpenoids, PFUA, among others, may help prevent aging and NDDs [25]. Caloric restriction and regular physical activity may also help promote brain function and prevent certain disorders.

#### 1.1.5. Medical Factors

Both AD and PD are associated with a wide range of medical factors that increase the risk of occurrence. Hypertension, high cholesterol, hyperhomocysteinemia, smoking, obesity, infectious environments, excessive alcohol consumption, chronic anemia, traumatic brain injury, and depression are considered significant drivers, along with environmental exposures, for AD [2]. In contrast, in PD, high iron intake, chronic anemia, and traumatic brain injury are identified as significant risk factors. Conversely, hyperuricemia, smoking, and coffee consumption are associated with a lower risk of PD [26]. These medical factors interact in complex ways with genetic predisposition and environmental exposures, adding to the understanding of the multifaceted causes of these NDDs. In addition, aging, and medical conditions such as cardiovascular disease, obesity, and diabetes also increase risk. Cardiovascular diseases such as stroke, atrial fibrillation, and heart failure contribute to neural damage and affect cognitive function, underscoring the importance of a holistic approach to understanding and treating these complex conditions [14,27].

#### 1.1.6. Other Factors

According to studies, emotional status and addiction are important risk factors for NDDs. For example, sleep disorders such as insomnia are associated with an increased risk of NDDs, particularly in the early stages of AD, which may worsen as the disease progresses [2]. In addition, a growing body of research suggests that certain emotional features, such as anxiety and depression, may be associated with or influence the development of these diseases [28]. Smoking and alcohol also contribute to an increased risk of cerebrovascular disease because they produce free radicals, increase oxidative stress, and promote pro-inflammatory effects, all of which can cause additional brain damage [2]. These findings underscore the need to consider not only medical and environmental factors but also emotional and behavioral factors in understanding and preventing NDDs such as AD and PD. Although it is possible to track the development of NDDs using anthropometric, lifestyle, and clinical parameters, the underlying processes are still poorly understood [29,30].

## 2. Current Diagnostic Strategies

The effective treatment of NDDs relies on early detection, as it allows better management of symptoms and slower progression of the disease. Diagnostic methods include assessment of behavioral symptoms, biological fluid biomarkers, neuroimaging, brain conductivity and electrical activity, neuropathological tests, among others. Most NDDs have low specificity for individual diagnostics, which makes it costly and more difficult to determine an appropriate diagnosis [31,32,33].

### 2.1. Neuropathological Techniques

**Figure 3:**
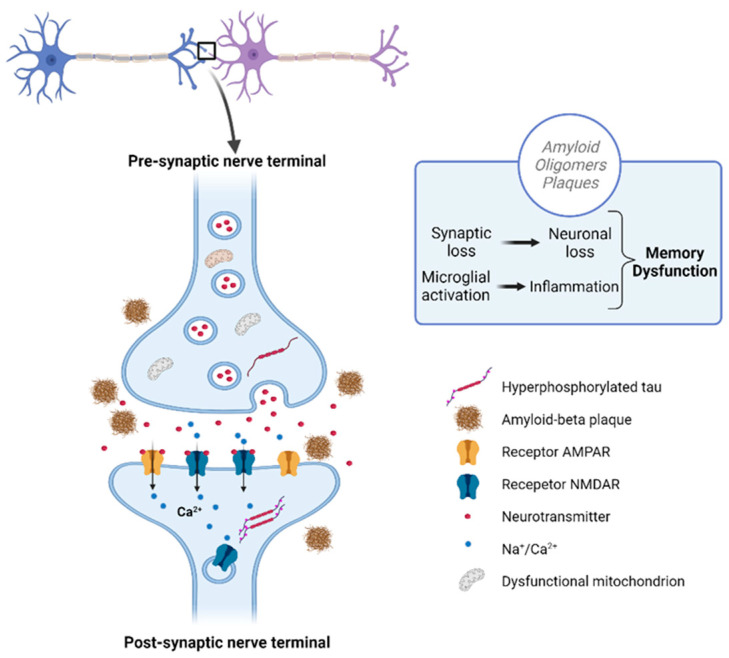
Disrupted communication in Alzheimer’s: Aβ plaques and tau tangles accumulating at the synapses disrupt communication and lead to synaptic loss. Created with BioRender.com.

At the initial stages of AD, the symptoms of memory impairment start to show up due to synaptic damage. This can be identified through the evaluation and measurement of synaptic protein activity, such as neurogranin, visinin-like protein-1, and synaptotagmin-1 (Figure 3). Synaptic loss results because of Aβ and tau accumulation at the synaptic sites. Defects in axonal transport, mitochondrial damage, and oxidative stress may also be used as identifiers of synaptic loss [3,31,34]. Besides influencing synaptic loss, the accumulation of Aβ also influences cholinergic neurotransmission, and decreases choline reception and the release of acetylcholine. Acetylcholine has a key role in cognitive function, being involved in many physiological processes such as sensory information, learning, attention, and memory [14].

### 2.2. Psychological and Neuropsychological Assessment

Psychological and neuropsychological methods are used to evaluate cognitive deficits, observe disease progression, and verify treatment effectiveness. These include short-term memory assessments (e.g., the Visual Short-Term Memory Binding Test) and executive function assessments, such as the Wisconsin Card Sorting Test and Delis–Kaplan Executive Function System, which detect NDDs and vascular dementias. In turn, these correlate with pathological changes found in patients and allow a cost-effective and non-invasive approach to monitoring disease progression. The score obtained from the Alzheimer’s disease cooperative study-preclinical Alzheimer cognitive composite (ADCS-PACC) scale, which comprises both memory and executive function tests, was found to be correlated with β-amyloid levels in patients’ cerebrospinal fluid [33].

### 2.3. Connectivity and Electrical Activity

Transcranial magnetic stimulation is a non-invasive method that allows the assessment of intracortical circuits which depend on various neurotransmitters and has been reported to have some potential for differential diagnosis of NDDs. However, when it comes to the electrical activity of the brain, the use of electroencephalographic patterns has yet to improve the accuracy in diagnosing NDDs [33].

### 2.4. Neuroimaging

Neuroimaging, including magnetic resonance imaging (MRI) and positron emission tomography (PET), makes it possible to determine and stage NDDs. While MRI can identify pathological changes (e.g., damage to white matter and alterations of brain volume) and help differentiate them from regular aging, PET can assess metabolic activity (e.g., glucose metabolism) and measure Aβ and tau protein levels [33,35]. AD has a characteristic pattern of atrophy in several structures of the medial temporal lobe. Several imaging techniques are used to evaluate biomarkers for AD: MRI, the recommended first step following clinical evaluation, which can also be used to evaluate cerebral microbleeds that result from cerebral amyloid angiopathy; F18-fluorodeoxyglucose-positron emission tomography (F18FDG-PET), used for differential diagnosis of NDDs, to identify the extent and localization of neurodegenerative processes and predict short-term clinical profile; amyloid-PET, the most useful for ruling out AD, as it allows the visualization of Aβ plaque accumulation, increasing diagnosis precision, and permits real-time monitoring of β-amyloidosis; and at last, tau-PET, an important biomarker for differential diagnosis between AD and other neurodegenerative tauopathies, which can track the progression of the disease, as well as the spreading of tau along brain networks, and therefore helps to better understand the interaction between tau and Aβ [3,32,34,36,37].

### 2.5. Biomarkers from Biological Fluids

**Figure 4:**
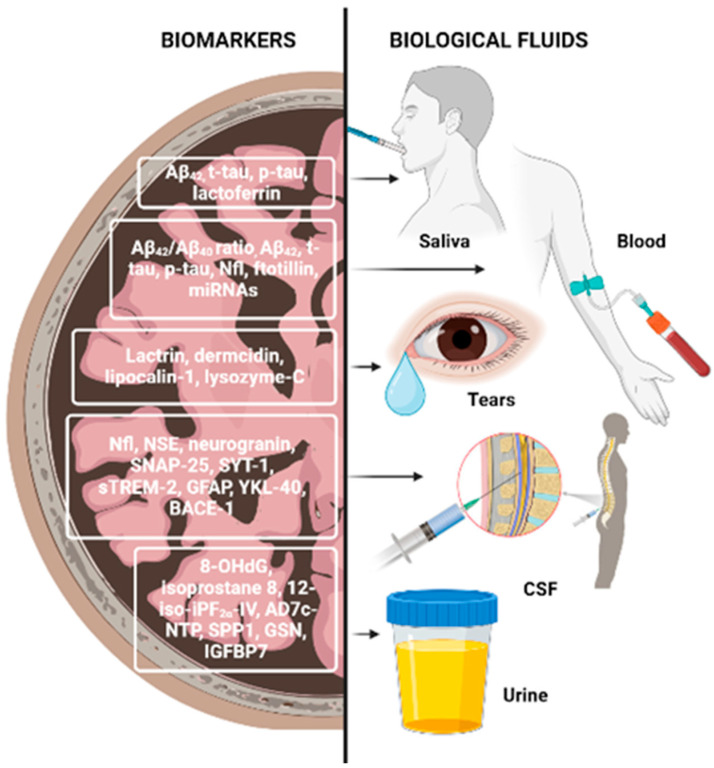
Neurodegenerative disease biomarkers. Abbreviations: 8-OHdG—8-hydroxy-2′-deoxyguanosine; Aβ—amyloid-β; AD7c-NTP—Alzheimer-associated neuronal thread protein; BACE-1—βeta-site APP-cleaving enzyme 1; GFAP—glial fibrillary acid protein; GSN—gelsolin; IGFBP7—glial fibrillary insulin-like growth factor-binding protein-7; NfL—neurofilament light chain protein; NSE—neuron-specific enolase; SNAP-25—synaptosomal-associated protein 25; sTREM-2—soluble triggering receptor expressed on myeloid cells 2; SYT-1—synaptotagmin-1; YKL-40—chitinase-like protein. Created with BioRender.com.

Biomarkers can be detected in the cerebrospinal fluid (CSF) or plasma years before symptoms emerge; therefore, early screening for NDDs is essential [35]. CSFs are the most reliable biological fluids for the evaluation of biomarkers associated with central nervous system disorders, allowing follow-ups on neurodegeneration progress. In this type of matrix, it is possible to identify a differential range of molecules involved in pathological mechanisms. Molecules that can be used as NDD biomarkers include the following: amyloid-β (Aβ) and tau proteins (mainly used for AD diagnosis), which confer high accuracy and can distinguish AD from other types of dementia; α-synuclein, a protein that regulates synaptic vesicle trafficking, found at lower levels in patients with PD, or decreased in its oligomeric and phosphorylated forms; neurofilament light chain (NFL), a scaffold protein found in the neuronal cytoskeleton, the concentration of which increases after axonal injury, in patients with AD, atypic PD, multiple sclerosis, and others; neuron-specific enolase, a glycolytic enzyme present in axoplasmatic transport, neuronal energy metabolism and cell survival, representing a marker of neuronal damage and found significantly increased in AD; neurogranin, a post-synaptic protein that plays an important role in learning and memory by maintaining long-term potentiation and synaptic plasticity, which serves as a biomarker for dendritic instability and synaptic generation and appears to be specific for AD; and synaptosomal-associated protein 25 and synaptotagmin-1, two synaptic proteins which are promising biomarkers for synaptic damage and loss. Besides these, several biomarkers of glial activation were proposed in recent studies, seen in Figure 4. For example, the receptor expressed on myeloid cells 2 has an important role in microglia, being involved in cytokine release, proliferation, and APOE binding and shielding of Aβ plaques. Furthermore, its soluble domain of the receptor expressed on myeloid cells 2 is closely associated with tau-related neurodegeneration, and it is suggested to be non-specific for AD, but useful for detecting a microglia response induced by any neuronal damage. Glial fibrillary acid protein (GFAP) is another marker of glial activation, activated and released by astrocytes during neurodegeneration. Moreover, chitinase-like protein (YKL-40) is a microglial and astrocyte marker, whose concentration increases with AD progression (however, it is not specific for AD) [32,33,34].

Despite the blood–brain barrier limiting the transportation of markers to systemic circulation, blood represents a more accessible biological fluid when compared to CSF. Additionally, the decreased ratio of Aβ42/Aβ40 found in plasma correlates with CSF levels and Aβ deposition in the brain, and tau protein levels can also be correlated as such, as higher concentrations in the blood indicate a higher concentration in the brain. NFL can also be measured in blood as a reliable biomarker for screening neurodegenerative processes and monitoring disease progression and therapy. Plasma GFAP was found to increase in early AD, and its levels were more accurate than in CSF. Specifically, it measures astroglial damage, as it can help detect reduced myelin and axonal changes. Epigenetics has an important role in biomarker discovery due to microRNA’s ability to regulate gene expression. Most dysregulated microRNAs are related to molecular mechanisms associated with AD pathogenesis (e.g., inflammation, apoptosis, Aβ, tau signaling pathways), however, this approach has some limitations [32,34].

Saliva is an interesting biomarker for NDDs due to it being non-invasive, cost-effective, and particularly because changes in saliva composition arise with aging and so could also be related to neurodegeneration development. Central nervous system target proteins have been reported to be excreted through saliva. While Aβ levels faced controversial results, the p-tau/t-tau ratio was found to significantly increase in AD patients; oligomeric α-synuclein/total α-synuclein could be important in defining PD progression; heme-oxygenase-1 (HO-1) and AChE activity were reported to be increased in PD patients’ saliva and therefore are possible candidates as biomarkers for PD; lactoferrin, an antimicrobial peptide showing similar binding properties to Aβ, was also suggested as a salivary biomarker as it has been detected in plaques, NFTs and microglia of AD patients’ brains. It is present in human saliva in high quantities and was detected at low levels in patients with AD and amnestic mild cognitive impairment, with a sensitivity and specificity of 100%. Conversely, it was found at high levels in PD patients. Furthermore, it was also reported there was a positive correlation between lactoferrin and CSF Aβ_42_, t-tau, and mini-mental state examinations. Despite this, saliva is affected by several factors and its biomarker levels are prone to fluctuation [32,38].

Tears have also been suggested as a potential biomarker of NDDs, as changes in the eye’s microenvironment promote differences in tear proteins, and Aβ plaques have been found in the retina and lens of AD patients [32].

Urine, on the other hand, is easily normalized and represents a good biological fluid as a source of biomarkers for the diagnosis and monitoring of renal dysfunctions. Oxidative stress and oxidative DNA damage play important roles in the early stages of AD, as reactive oxygen species (ROS) combined with mitochondrial and nuclear DNA leads to 8-hydroxy-2′-deoxyguanosine (8-OHdG) production, and that can be used to monitor cellular dysfunction in urine. Isoprostane 8, 12-iso-iPF2α-IV results from arachidonic acid peroxidation by free radicals and is found elevated in AD patients. Urinary Alzheimer-associated neuronal thread protein is a transmembrane phosphoprotein that co-localizes with NFT, and it is associated with tau accumulation in AD patients. In recent studies, three differentially expressed proteins were identified in the urine of AD patients: secreted phosphoprotein 1, involved in modulation of macrophage immunological profile for a better ability to mediate Aβ clearance; gelsolin, which binds to Aβ, solubilizes its fibrils and inhibits fibrillization in the brain; and insulin-like growth factor-binding protein-7, which is a key mediator of memory function and contributes to cell homeostasis [32].

## 3. The Potential of Secondary Bioactive Metabolites from Plant-Based Food as Theravention Agents against NDDs; Their Action Mechanisms and Bioavailability

### 3.1. Polyphenols

Polyphenols, one of the most abundant classes of SBMs in plant foods, are emerging as a promising approach for the development of effective agents for the prevention of NDDs due to their neuroprotective effects [39]. This chemical family includes flavonoids (e.g., epigallocatechin, epigallocatechin gallate, catechin, quercetin, luteolin), non-flavonoids (e.g., caffeic acid, gallic acid, ferulic acid, syringic acid), and anthocyanins (e.g., malvidin), which are pharmacologically important SBMs [40,41]. Dietary polyphenol supplementation is considered an appropriate therapeutic strategy to prevent or delay NDDs due to their neuroprotective effects, availability, and safety [42,43]. Studies suggest that polyphenols exert protective effects on the central nervous system, improving cognitive function and mitigating/reducing neuroinflammation, oxidative stress, neuronal death, and neurodegeneration in animal models and clinical trials. Although the exact pathways of these therapeutic benefits are still unknown, ongoing research suggests the potential of polyphenols as effective agents in the treatment of neuroinflammation and NDDs. These SBMs seem to act through various mechanisms related to cellular signaling and antioxidant responses that help reduce the production of reactive oxygen species (ROS), attenuate the accumulation of neuropathological markers (e.g., Aβ1–42, tau protein), reduce neurotoxin-mediated neuronal damage, reduce inflammatory mediators and neuroinflammation, modulate cholinergic function, improve mitochondrial function, promote proper autophagy, among others [42,44,45,46,47]. In addition, these SBMs activate nuclear erythroid 2-like factor 2 (Nrf2), a key regulator of antioxidant genes, and may affect the gut–brain axis and have anti-inflammatory properties by blocking the NF-kB pathway [45,47]. For instance, polyphenols have been shown to possess cell-protective properties against oxysterols, products of cholesterol oxidation that cause cell damage, oxidative stress, inflammation, Aβ accumulation, and mitochondrial dysfunction, associated with NDDs like AD and PD [48].

Accordingly, in an in vitro study by Yammine et al. [49], polyphenols like resveratrol, quercetin, and apigenin were able to prevent 7-ketocholesterol (7KC) cytotoxicity in neuronal N2a cells. This was achieved by attenuating oxidative stress and apoptosis, which consequently prevented autophagy activation, efficiently inhibiting 7KC-induced oxiapoptophagy. Mechanisms that reduce oxidative stress include a decrease in ROS production both in whole cells and at the mitochondrial level; attenuation of catalase levels and activity; upregulation of glutathione peroxidase 1 (GPx1) levels and activity; regulation of SOD1 and SOD2 levels and activity; and upregulation of Nrf2. Furthermore, these polyphenols prevented organelle dysfunction by increasing mitochondrial biogenesis. It is suggested they counteracted the loss of mitochondrial membrane potential (ΨΔm) and prevented decreased levels and expression of AMPKα, sirtuin 1 (SIRT1), and peroxisome proliferator-activated receptor γ coactivator-1α (PGC-1α), which are involved in mitochondrial biogenesis. In addition, they also increased protein and mRNA levels of peroximal mass marker ATP-binding cassette subfamily D member (ABCD), and downregulated peroximal biogenesis (Pex13, Pex14) and peroximal β-oxidation (Abcd1, Acox1, Mfp2, Thiolase A) genes. 7KC-induced oxiapoptophagy can therefore be prevented more efficiently in pre- and co-treatment by resveratrol, quercetin, and apigenin, which indicates the importance of polyphenols in preventing NDDs, especially apigenin, given its lower toxicity and neurotrophic activity [49].

On a separate note, an in vitro study revealed that phenolic extract (gallic acid, chlorogenic, caffeic acid, rutin, quercetin, kaempferol) of Senecio biafrae inhibited AChE and butyrylcholinesterase (BChE) activity, which is considered an effective strategy to treat AD as it prevents the breakdown of these enzymes in the brain and increases neurotransmitter levels at the synaptic cleft. Consequently, this would increase nerve cell communication, mitigating AD symptoms. It was also demonstrated that phenolic extract exhibited antioxidant and free radical scavenging activity through decreased NO production and Fe^2+^ chelation, the latter of which could attenuate hydroxyl radicals’ generation [50].

Accordingly, other studies reported the enzymatic inhibition of phenolic extracts against cholinesterase activity, suggesting its usefulness for the treatment of AD and other NDDs [51,52]. From the pool of polyphenols, gallic acid [53], caffeic acid [54], curcumin [55], apigenin [56], resveratrol [57,58], quercetin [59,60], and epigallocatechin gallate [61,62] are some that demonstrated an effective effect on pro-inflammatory gene expression. For instance, phenolic-rich maple syrup extract in a transgenic mice model of AD reported a decrease in AD-risk-associated inflammatory protein expressions, such as the suppressor of cytokine signaling-6, the triggering receptor expressed on myeloid cells 2, and the stimulator of interferon genes TMEM173 (transmembrane protein 173) [63]. On another note, an in vivo study on polyphenolic extracts of scope grape seed (GSPE) reported neuroprotective effects on age-related NDDs through microbiota metabolism, which is known to produce phenolic acids from polyphenols. Thus, following GSPE treatment, rats had increased levels of two phenolic acids ([3-(3-hydroxyphenyl) propionic acid and 3-hydroxybenzoic acid]) in the brain, capable of intervening in Aβ accumulation and aggregation and inhibiting assembly of neurotoxic Aβ peptides, preventing NDDs [64]. Recent studies have demonstrated the potential of curcumin, an active hydrophobic polyphenol extracted from the rhizomes of the plant *Curcuma longa* Linn belonging to the Zingiberaceae family, in the prevention of NDDs such as AD and PD [65]. Giacomeli et al. [55] observed that the administration of nanoencapsulated curcumin (1 or 10 mg/kg, orally) for 14 days is a promising approach for the application of a neuroprotective agent in the prevention and treatment of AD. Curcumin holds therapeutic promise due to its antioxidant, anti-inflammatory, and iron-chelating properties, as well as its interaction with α7 nicotinic acetylcholine receptors, as demonstrated by Nebrisi et al. [66]. These interactions improve neurobehavioral function and restore levels of neurotransmitters such as dopamine.

Apigenin is a natural flavone found in a variety of plant sources, including fruits, parsley, celery, spinach, olive oil, and chamomile [56], which has demonstrated the potential to reduce AD symptoms in transgenic flies expressing human Aβ42 in neurons [56] and in animal models of PD [67]. Apigenin administration attenuated the histopathologic changes induced by 1-methyl-4-phenyl-1,2,3,6-tetrahydropyridine in brain tissue and reversed the changes in expression and protein levels of pro-inflammatory cytokines such as tumor necrosis factor-alpha, interleukin-1-beta (IL-1β), IL-6, and transforming growth factor-beta.

Resveratrol is a well-known antioxidant found in a variety of foods such as grapes, wine, peanuts, and mulberries that has been extensively studied for its bioactivity and potential therapeutic relevance [58]. In terms of AD, resveratrol has demonstrated the ability to significantly reduce the cytotoxicity of Aβ1–42 peptides against human neuroblastoma SH-SY5Y cells. In addition, Al-Edresi et al. [57] proposed a new mechanism of action for resveratrol, which may activate some genes associated with slower aging and increased longevity, thus preventing DNA oxidation in cells. Regarding PD, studies in animal models (adult female BALB/c mice, 10 weeks old) suggest that resveratrol improves brain function and controls inflammatory processes, protecting dopaminergic neurons, and reducing neuroinflammation and apoptosis through the Akt signaling pathway [68]. In addition, Liu et al. [68] demonstrated the ability of resveratrol to synergize with low doses of l-3,4-dihydroxyphenylalanine (L-DOPA), the main drug for PD, potentially reducing the required dose and improving treatment outcomes.

Quercetin is a flavonoid found in a diversity of samples, such as wine and tea, and is mostly found as a glycoside in higher plants in the form of isoquercetin, rutin, and hyperin [69]. Several researchers have shown that the administration of quercetin and its derivatives promotes the inhibition of secretase, AChE, BChE, histone deacetylase, and tyrosinase enzymes [59,70]. Chang et al. [54] investigated the mitigating effect of caffeic acid on AD pathogenesis and related mechanisms in high-fat diet-induced hyperinsulinemic rats. The data obtained showed a decrease in the expression of phosphorylated tau protein and attenuation of the expression of amyloid precursor protein (APP) and β-site APP cleaving enzyme in the hippocampus of rats treated with caffeic acid. Another study evaluated the effect of gallic acid in the transgenic AD mouse model of mutant human amyloid β-protein precursor/presenilin 1 (APP/PS1) and found that gallic acid attenuated cerebral amyloidosis, including brain parenchymal and cerebral vascular β-amyloid deposits, and reduced cerebral amyloid β-proteins [53].

The use of polyphenols in nutraceuticals has been delayed because many of these substances have poor oral bioavailability. To overcome this obstacle, bio-based polymers are suitable delivery systems. They are biocompatible, biodegradable, resource-sustainable, and nutritionally valuable [71,72]. Polyphenols are recognized as xenobiotics after ingestion. That is why their bioavailability is very low compared to micro and macronutrients. The bioavailability of these compounds depends on their class. This is due to their structure, composition, and the metabolites they can form. Once ingested, the less complex phenolic compounds can be hydrolyzed and bio-transformed into hydrophilic conjugated metabolites that enter the bloodstream, are distributed to organs, and are excreted in the urine [71]. The intestinal microbiota is responsible for the extensive degradation of the parent compound into a series of low-weight molecules that can be absorbed. Consequently, it may be responsible for the beneficial effects associated with polyphenols rather than the compound itself. The highest concentration found in the plasma is rarely more than 1 µM, and the peak usually appears 1–2 h after ingestion [71]. Lorenzo and collaborators [73] reviewed the human bioavailability of polyphenols. They focused on anthocyanins, which are the most abundant polyphenols in food with an average concentration of 115 ± 259 mg/100 g. They are found in black elderberries (1316 mg/100 g), black chokeberries (878 mg/g), and black currants (595 mg/100 g), as well as in red wine, colored beans, and vegetables. However, they are very poorly bioavailable, averaging 1–2% when ingested. Several factors such as food matrix or technological/processing conditions, enzymatic patterns, and microbiota composition affect the bioavailability of anthocyanins [73,74].

Regarding the clinical use of these polyphenols, the daily use ranged from 2.1 to 94.47 mg, and the food matrix generally contains blackcurrant and orange juice. However, they did not show any improvement in vascular reactivity or reduction in oxidative stress, not only because of the degradation at the gastrointestinal level, but also because of the short period of treatment. In urine, the percentage varied within 0.79 ± 0.9% of the ingested dose [73]. Another example is quercetin, which is found in cranberries (149 mg/100 g), onions (65 mg/100 g), green tea, and red wine (2.5 and 1.6 mg/100 mL, respectively). Flavonol intakes ranged from 16.7 to 400 mg/day, but studies did not show any changes in cardiovascular parameters or oxidative stress. The highest plasma concentration was 144 ± 12.3 nM. However, when mediated by collagen, quercetin provided by food such as onion soup or supplements (138 and 150 mg quercetin, respectively) was shown to be effective in inhibiting platelet aggregation [73,75]. The bioavailability of polyphenols is very low when provided from a food source. However, the beneficial effects of these compounds make them a good candidate for pharmaceutical purposes, due to their anti-microbial, anticancer, and antioxidant properties. To solve their bioavailability issue, nanomaterials with a delivery system can be used to take the compound to the target tissue, increase the amount of compound ingested, or finally, isolate the compound of interest into a supplement pill [71,72].

### 3.2. Polyunsaturated Fatty Acids

The importance of PUFAs, particularly those of the omega-6 (ω-6) and omega-3 (ω-3) families, lies in their potential therapeutic applications and their role as key SBMs of the diet. Epidemiological evidence links a deficiency of specific PUFAs such as linoleic acid (LA, ω-6), eicosapentaenoic acid (EPA, ω-3), and docosahexaenoic acid (DHA, ω-3) to an increased risk of chronic diseases such as cancer, cardiovascular disease, and diabetes [39]. In particular, ω-3 PUFAs, commonly found in fish and fish oil, have biological properties that may be beneficial in NDDs. On the other hand, ω-6 PUFAs, especially arachidonic acid, can cause neuroinflammation and neuron damage in excessive amounts. It is suggested that an optimal ratio of omega-3 to omega-6 is needed to diminish the risk of NDD development [39,76].

Supplementation with ω-3 PUFAs has been shown to improve neurogenesis, executive function, and learning in animal models. Conversely, its deficiency has been associated with memory impairment and reduced hippocampal plasticity [77]. Particularly, EPA has been demonstrated to enhance the activity of two neurotrophic factors essential for neuronal survival, differentiation, and plasticity, which are the brain-derived neurotrophic factor and glial cell line-derived neurotrophic factor, through epigenetic mechanisms such as methylation and hydroxymethylation modulation of these genes, which in turn reduced the toxic effect of neurotoxin 6-hydroxydopamine (6-OHDA) [78]. Furthermore, ω-3 PUFAs also exhibit anti-inflammatory properties by reducing microglial activation associated with aging and oxidative stress, as well as increasing levels of pro-inflammatory cytokines. These properties highlight their potential to mitigate AD [79]. A study by Yan et al. [80] also reported ω-3 PUFAs’ ability to promote brain Aβ clearance by restoring expression of lipoprotein receptor-related protein 1 (LRP-1), an efflux transporter of the blood-brain barrier. Moreover, in vivo and ex vivo studies using animal models of aging and NDDs after treatment with PUFAs demonstrated their potential to prevent AD and PD [46,80].

There are three main pathways from which PUFAs are metabolized: lipoxygenase (LOX), cyclooxygenase (COX), and cytochrome P450 (CYP). As a result, oxidized lipid mediators (oxylipins) are produced and play a critical role in modulating the inflammatory response by influencing gene expression and cell membrane composition. Furthermore, it has been reported that in vitro manipulation of cell membrane PUFA composition alter the function and/or signaling of various receptors such as cholinergic, dopaminergic, and GABA receptors. While oxylipins generated from LOX and COX exert neuronal excitatory effects, CYP-generated mediators exhibit neuroprotective effects. Moreover, it has been reported that inhibition of these metabolites’ degradation by soluble epoxy hydrolase had beneficial effects on AD and PD and could protect dopaminergic neurons against neurotoxins in vivo. Thus, soluble epoxy hydrolase could be an important target for NDDs involved in PUFA metabolism. CYP PUFA metabolite levels depend on endogenous levels of its PUFAs’ precursors; however, they are synthetized in small quantities in humans. As such, they largely depend on exogenous uptake; therefore, a diet rich in these fatty acids is of great importance for NDDs. Despite PUFA metabolites’ mechanisms of action remaining unclear, Ep-PUFAs (derived from CYP pathway) appear to be able to mitigate neuroinflammation through increasing anti-inflammatory and decreasing pro-inflammatory cytokines’ production, which is essential for NDDs [81]. In terms of PUFA bioavailability, our focus will be on the ω-3 fatty acids eicosapentaenoic (EPA) and docosahexaenoic (DHA) that are known to have numerous wellness benefits such as those anti-cardiovascular, anti-inflammatory, anti-depressant, antioxidant, anti-hypertensive, among others. Plant sources such as chia seeds, flaxseeds, walnuts, or vegetable oils such as canola oil, soybean oil, flaxseed oil, as well as animal sources such as tuna, salmon, and sardines, are rich sources of this compound [82].

The lipid form, as well as its structure and food matrix, affects the bioavailability of FAs. After a study that evaluated 14 weeks of supplementation with algae oil (2.4 g/d DHA; EPA in a ratio of 2.7:1) and with fish oil (2.0 g/d DHA; EPA in a ratio of 0.7:1), it was verified that algae oil increases the plasma levels of DHA, and fish oil increases the levels of EPA. On the other hand, supplementation with 600 mg/d of DHA from either fish or algae oil capsules for 2 weeks showed a significant increase in plasma DHA levels of 71.60–84.22 μg/mL in the vegetarian/vegan group, starting from a baseline of 34.10 μg/mL, compared to the omnivore group [83]. Nana et al. [84] evaluated the results after 88 patients took two soft gel capsules each containing 481 mg EPA triglyceride and 481 mg DHA triglyceride concentrates (500 mg/g) twice daily for 28 days, providing a daily dose of 1 g of EPA and DHA-free fatty acid equivalents, respectively. They observed that the bioavailability of EPA and DHA in ileostomy fluid was increased. DHA and EPA are essential ω-3 fatty acids that our bodies cannot make on their own. Aquiring them from n-3 PUFA-rich foods such as fatty fish or supplements can significantly improve health by supporting brain function, reducing the risk of heart disease, lowering bad cholesterol, managing inflammation, and potentially improving mood [82,83].

### 3.3. Proteins and Amino Acids

NDDs such as AD and PD are characterized by abnormal protein aggregation resulting from the polymerization, misfolding, and subsequent aggregation of one or more peptides or proteins [85]. Recent studies have highlighted the critical role of proteins and amino acids in the prevention and treatment of these diseases, with specific proteins directly implicated in their pathogenesis. In AD, errors in the processing of APP in the brain lead to the production of a short fragment of APP known as Aβ. The accumulation of Aβ peptides in the brain, which lose their native structure and take the form of extended parallel beta sheets, triggers the destruction of nerve cells, causing AD. In PD, aggregation of the protein α-synuclein, which can form different types of aggregates, plays a central role in the pathogenesis. The abnormal aggregation and accumulation of this protein in the brain leads to the formation of intracellular inclusions in Lewy bodies, which can cause neuronal dysfunction and cell death, and trigger inflammatory processes that contribute to disease progression [46,86]. Therefore, therapeutic strategies to prevent protein aggregation and reverse the damage caused have been a focus of research. One example is acetyl-L-carnitine, a natural compound with potential neuroprotective and neurotrophic effects, whose mechanisms of action include the ability to modulate metabolic processes, reduce oxidative stress, and promote synaptic plasticity, highlighting it as a possible therapeutic agent which could improve brain function and mitigate the negative effects associated with these disorders [86]. A recent study demonstrated that the 14-3-3 protein influences Aβ fibril production and interacts with other critical proteins in AD. Modulation of this protein may have therapeutic applications in AD, particularly as a biomarker for early detection and prognosis [87]. In addition, chaperone proteins such as Hsp70 and Hsp90 have been identified as important modulators of neurodegeneration for protecting neuronal integrity [88,89]. These proteins can assist in the proper folding of proteins, prevent their aggregation, and promote their degradation when necessary [89]. Dietary or therapeutic interventions aimed at increasing the expression or activity of these chaperone proteins may therefore represent a promising strategy for the treatment or prevention of NDDs.

Understanding amino acid bioavailability is becoming increasingly important with the growing popularity of plant-based diets. The bioavailability of amino acids refers to the proportion of dietary amino acids that are absorbed and utilized by the body. Several factors influence the body’s response to protein synthesis following the ingestion of protein, including the amount and, more importantly, the quality of the protein ingested, particularly its amino acid profile. Traditionally, due to their generally higher bioavailability, animal proteins have been considered the gold standard for protein quality. However, as people look for alternative ways to meet their protein needs, research into plant-based protein sources is booming [90,91]. The literature reports that in vegetables the indispensable amino acid bioavailability ranged around 63–74%, whereas the value is around 90% for meat and eggs [92,93]. Additionally, the treatments applied to food are also a determinant of bioavailability. For example, the process of cooking, such as boiling or grilling, bovine meat could vary the bioavailability by 2.5% [94]. On the other hand, in pork, it is not verified, nor in the smoking process of bacon. However, the process of curing improves the bioavailability in ham from 95 to 99% [95]. In the case of heat treatment applied to nuts, such as roasting, protein digestibility is affected by 10% [96]. An understanding of the bioavailability of amino acids is essential, especially for the optimization of protein intake in plant-based diets. However, protein digestibility can be significantly affected by seemingly minor food processing techniques, from roasting nuts to curing meats.

## 4. Plant-Based Foods and Herbs Effective against NDDs

NDDs have been treated with botanicals since as early as 6000 BC in regions such as India, China, Africa, and pre-Columbian America. The Incas and Aztecs, for example, used more than 1500 plants to treat NDDs. These included *Ficus platyphylla*, *Gladiolus dalenii* and *Voacanga Africana*. The potential of natural products as a source of therapeutic agents is highlighted by the historical use of plants and their extracts in traditional medicine. Plants are a rich source of both primary and secondary metabolites, many of which have a variety of pharmacological benefits. Compared to isolated compounds, these natural products often contain many SBMs, providing a broader spectrum of activity. The isolated compounds themselves are promising, possessing unique structures with biochemical specificity, chemical diversity, and other favorable molecular properties. These characteristics make them ideal lead structures for the discovery of new herbal medicines. The synergistic effect of the multiple components of herbal remedies is one of the perceived advantages of these compounds over traditional allopathic medicine. These multiple components work together to potentially combat disease more holistically. In addition, further benefits in terms of improved absorption and efficacy in the body can be achieved through the incorporation of herbal extracts into novel drug delivery systems [97,98].

Concerning AD, a growing body of research, including epidemiological studies, clinical trials, and long-term population surveys, suggests a positive association between daily consumption of plant-based foods and medicinal plants and a reduced risk of the disease, based on observed outcomes such as reduced tau phosphorylation, decreased amyloid aggregation, improved memory function, reduced oxidative stress, and reduced neuronal loss, seen in Table 1 [99,100,101,102,103,104]. According to research by Loureiro et al. [100], a concentrated extract from grape seeds and skins (40 μM) significantly inhibited the formation of amyloid-β peptide (Aβ1–42). This fragment is thought to play an important role in the progression of AD. The extracts showed impressive inhibition rates, nearly 97% for grapes. Thuphairo et al. [101] investigated the potential of green pepper extract against AD-associated enzymatic pathways. They found that green peppers had the strongest antioxidant and inhibitory effects against enzymes involved in AD development, including BChE (butyrylcholinesterase) and BACE1 (β-secretase).

On the other hand, yellow pepper showed the highest level of acetylcholinesterase (AChE) inhibition. The biological activity of various South African medicinal plant extracts against AD was investigated by Thakur et al. [104]. They found promising results with leaves from certain plants, including *Xysmalobium undulatum* (*Apocynaceae*), *Cussonia paniculata* (*Araliacea*), and *Schotia brachypetala* (*Fabaceae*). These extracts significantly reduced the production of Aβ1–42 by 77, 58, and 45%, respectively. Tripathi and Mazumder [102] reviewed the potential benefits of apple cider vinegar for AD. Their review highlights the presence of several SBMs, including flavonoids, phenolic compounds, minerals, and vitamins. Vitamins with high antioxidant potential, such as vitamin E, may help combat the free radicals that contribute to cell damage in AD. In addition, the phenolic compounds in cider have antioxidant and anti-inflammatory properties that may help prevent memory decline through multiple cellular pathways. Tzekaki et al. [103] investigated the implication of BMI1 in Alzheimer’s disease and the possibility of reversing the onset of the disease by administering extra virgin olive oil (EVOO) for an effect in patients with mild cognitive impairment (MCI). The potential role of BMI1, a neuroprotective protein, in AD was the focus of their study. The study included 12 months of treatment with the EVOO supplement. Following treatment, researchers observed significantly higher BMI1 levels in EVOO-treated patients compared to controls (*p* < 0.001). A decrease in p53 levels, which were higher in the control group, was associated with this increase in BMI1. In addition, patients treated with EVOO showed a restoration of the Aβ1–42/Aβ1–40 ratio. This finding suggests a potential beneficial effect of EVOO in ameliorating AD by facilitating Aβ clearance and reducing toxic aggregation.

Regarding PD, a growing body of research, including animal and clinical studies, suggests a positive association between daily consumption of plant-based foods, medicinal plants, and their constituents with a reduced risk of the disease. This is based on observed outcomes such as the inhibition of dopamine metabolizing enzymes, reduction of oxidant markers, increasing of antioxidants, and suppression of neuroinflammation [121,127,128,133,136]. Here are some specific examples that demonstrate the promise of plant-based approaches: Abdel-Sattar et al. [120] evaluated the antiparkinsonian activity of 80% methanolic extracts from seeds and sprouts of Egyptian *Vicia faba* L. in comparison to L-DOPA in rotenone-parkinsonian mice. Rotenone was administered in small amounts involving nine subcutaneous injections at 1 mg/kg per 48 h, along with as seed extracts which were given at 200, 400, and 600 mg/kg per day. The experiment showed that the extract at a concentration of 600 mg/kg showed significant antioxidant, anti-inflammatory, and neuroprotective effects. The neuroprotective activity may be related to the content of SBMs such as flavonoids, phenolic acids, and others. The Nicholatos research group [128] investigated a complex relationship between Sirtuin 6 (SIRT6), a stress-responsive protein encoded by the SIRT6 gene, and both tobacco use and PD. SIRT6 is abundant in the brains of PD patients but is decreased in the brains of tobacco users. Interestingly, in vitro assays using a high concentration (200 μg/mL) of nicotine suggest that nicotine may suppress SIRT6 conversion, potentially increasing neuronal resistance to cell death. However, it is important to note that this was an in vitro study and the safety and efficacy of such high doses of nicotine in humans remains unclear. In addition, the well-documented adverse health effects of tobacco use, including increased risk of cancer, heart disease, and lung problems, far outweigh any potential benefits associated with SIRT6. Yu and colleagues [127] investigated the effects and mechanisms of ginkgo biloba dropping pill (GBDP) in the treatment of PD. For in vivo assays, 50 mg/kg of GBDP was administered daily, while for in vitro assays they used 60, 90, and 120 mg/mL for both leaf extract and GBDP. The results indicated that GBDP protected dopaminergic neurons against 6-OHDA- and MPTP/MPP^+^-induced neurotoxicity, which may be mediated by Akt/GSK3β signaling pathways. The research team of Lei et al. [121] found that safflower flavonoid extract (SAFE) could bind to the PD-related protein DJ-1. They also isolated a standardized SAFE and prepared drop pill. SAFE may be involved in behavioral, biochemical, and pathological changes in 1-methyl-4-phenyl-1,2,3,6-tetrahydropyridine-induced PD mice and rotenone- or 6-OHDA-induced PD rats. To evaluate this, they experimented with three doses of SAFE, 25, 50, and 100 mg/kg in 20 mice each. They concluded that the extract exerts neuroprotective effects on 6-OHDA-induced dyskinesia and dopaminergic neuron degeneration in PD mice, as well as reduces the secretion of inflammatory factors via attenuation of microglial NLRP3 inflammasome activation, suggesting that SAFE could be a potential drug for PD treatment. *Hypericum perforatum* is a medicinal herb used in the treatment of various mental disorders. Kumar et al. [133] focused on the antioxidant changes in MPTP-induced PD. *Hypericum perforatum* extract was administered orally to mice at 300 mg/kg b.w. and injected at 30 mg/kg b.w. for 5 consecutive days. The study concluded that these extracts can reduce oxidative stress, ameliorate ultrastructural changes in the brain tissue of PD models, and provide neuroprotection against MPTP induced in PD models. The historical use of herbal remedies for NDDs is extensive. Most of these investigations were focused on the anti-inflammatory and antioxidant properties of SBMs (e.g., polyphenols, terpenoids, carotenoids, alkaloids) found in medicinal plants and food products. However, further research is needed to definitively establish their efficacy and optimize their use. To build on this rich tradition and unlock the full potential of herbal solutions for NDDs, modern clinical trials and scientific research are needed.

## 5. Future Perspectives

NDDs are prevalent chronic disorders that are currently being extensively researched due to their rising prevalence and significant impact on the senior population. Alzheimer’s disease and Parkinson’s disease are among the most common NDDs. Alzheimer’s disease is primarily characterized by progressive memory loss, diminished cognition, and changes in behavior, which together lead to emotional instability and eventual mortality. Similarly, Parkinson’s disease is marked by motor symptoms such as tremors, rigidity, and bradykinesia, alongside cognitive decline and behavioral changes, and also leading to severe disability and increased mortality. The growing incidence of these diseases underscores the urgent need for improved diagnostic methods and effective therapeutic interventions. In this sense, the therapeutic potential of the main SBMs identified in herbs and food-related products has positive impacts on patients by slowing the progression of NDDs, according to scientific epidemiological research. They can decrease AChE activity, corticosterone levels, Aβ and tau-protein accumulation and aggregation, and cognitive impairments including in learning and memory [59,101,111,113,116,119]. By targeting these pathways, SBMs from plant-based foods and herbs hold promise as complementary therapies for managing neurodegenerative diseases, providing a multi-faceted approach to slowing disease progression and improving patient outcomes, due to their antioxidant, anti-inflammatory, and anti-apoptotic properties which may play a role in their mechanisms of action. However, the information related to their ideal dosages, absorption, variations across chemical forms, and potential interactions with other food components is currently limited.

According to the data collected, existing treatments help with some symptoms but have little impact on morbidity or death associated with NDDs. As a result of these shortcomings, more extensive research is needed to understand the characteristics of NDDs, their causes, and purported treatment strategies. A critical issue with current therapeutic approaches involving SBMs is their bioavailability. SBMs often face challenges such as rapid metabolism, low permeability, and decreased stability, which hinder their therapeutic efficacy. To address these limitations, scientific research suggests that utilizing nanotechnology and nanocarrier-based delivery systems can significantly enhance the delivery and effectiveness of SBMs derived from medicinal plants and food-related products [137,138,139]. Nanotechnology-based delivery approaches can boost the bioavailability of SBMs by enhancing stability, permeability, circulation time, and targeted distribution. By utilizing these enhanced delivery technologies, SBM therapeutic responses can be considerably increased, providing a viable route for creating more successful therapies for Alzheimer’s, Parkinson’s, and other neurodegenerative disorders.

## 6. Conclusions

NDDs profoundly impact the lives of individuals affected by them, since they cause progressive deterioration of nerve cells, leading to a range of cognitive, motor, and behavioral symptoms that significantly alter daily living and quality of life. The incorporation of plant-based foods rich in SBM offers a promising avenue for the prevention and management of NDDs. By leveraging the antioxidant, anti-inflammatory, and enzyme-inhibitory properties of SBMs such as terpenoids, polyphenols, and glucosinolates, it is possible to develop dietary strategies aimed at promoting brain health and mitigating the risk of NDDs. This approach not only promotes overall brain health, but also provides a natural and sustainable strategy to address the growing prevalence of NDDs. Further research is needed to fully understand the mechanisms and efficacy of these SBMs, but the potential benefits underscore the importance of a plant-based diet in maintaining cognitive health.

## Figures and Tables

**Table 1:** Effect of medicinal plants and food-related products on neurodegenerative diseases.

Disease	Food Source	Doses	Model	Outcomes	Ref.
AD	Apple leaves	200 and 400 mg/kg bw of extract for 9 days	Rats	The extract and phlorizin (main metabolite) exhibited strong antioxidant and BACE1 inhibitory activities	[105]
AD	Apple cider vinegar	10 µM of apple cider vinegar	high-cholesterol-fed rats	Reduced oxidative stress and memory impairment and shielded cholinergic hippocampus neurons from deterioration. Reduced tau phosphorylation and amyloid aggregation.	[102]
AD	Avocado oil	1 mL/kg bw	Wistar rats	The oil induced a substantial decline in neuronal loss in the CA1 and CA3 hippocampal regions.	[106]
AD	Banana peels	10 μL of plant extract (50–1000 μg/mL)	Swiss Webster mice	It was found that the extract may be used in therapies for memory impairments because of its antioxidant, AChE, and tyrosinase inhibition properties.	[107]
AD	*Carica* papaya leaves	100 µL of extract	male Wistar rats	The extract strongly reduced AChE and BChE activities	[108]
AD	*Elettaria cardamomum* extract	100, 200, and 400 mg/kg of extract for 8 weeks	T2DM rats	In diabetic rats, the extract prevented the accumulation of tau and amyloid. In the brains of T2D rats, the extract lowered AChE and caspase-3 activity.	[99]
AD	Grape leaves	100 mg/kg/day of grape leaf extract	Wistar rats	Treatment of AD rats with extract-enhanced brain function showed positive neurobehavioral changes. The neuromodulator effect of the extract was achieved through anti-amnesic activities against AlCl_3_-induced cerebral damages	[44]
AD	Grape seed	1 to 100 µM of extract over 24 h	TMHT mouse model	By neutralizing phospho-epitopes and upsetting fibrillary structure, the extract had substantial potential for therapeutic development for disintegrating paired helical filaments.	[109]
AD	Grape skin and grape seed	40 and 80 µM of extract	Model Human Blood–Brain Barrier	The production of Aβ(1–42) fibrils was significantly inhibited by grape extracts. The extracts had a stronger inhibitory impact than pure resveratrol.	[100]
AD	Green tea	400 mg/kg/mL/day for 8 weeks	Wistar rats	GT reduced antioxidant capacity and improved AChE activity.	[110]
AD	Lemon juice	0.6 and 1.2 mL/kg/day for 14 days	Rats	The results of this study showed that lemon juice may enhance cognitive function in rats with scopolamine-induced amnesia.	[111]
AD	*Lycopodiastrum* *casuarinoides*	10 µL of triterpenoids	Isolation of compounds and test for AChE and BuChE inhibitory activities.	The triterpenoids identified showed good inhibitory effects against AChE and BuChE.	[112]
AD	Medicinal plant extracts	50 µg/mL of extract for 8 h	In vitro screening	Of 33 plant extracts, 10 were determined to be active based on their capacity to considerably lower Aβ42 production.	[104]
AD	Medicinal plant extracts	10 μL of plant extract (15–150 μg/mL)	Human neuroblastoma cell lines	The plant extracts exhibited high inhibitory activity against AChE, BuChE, α– and β–Glc enzymes.	[113]
AD	*Melicope glabra* leaves	10 µL of alkylated quercetins	O-alkylated quercetins with selective AChE and β-secretase inhibitions	Influenced significant BACE1 inhibition.	[59]
AD	Mulberry fruit	100 mg/kg bw of extract for 1.5–3 weeks	APP/PS1 mice	The spatial memory and learning ability of APP/PS1 mice were significantly improved.	[114]
AD	Olive fruit	100 μg/mL of extract	SKN-1/NRF2 and HSP-16.2 in *Caenorhabditis elegans*	Reduction in oxidative stress and delay of Aβ induced paralysis due to the smaller presence of Aβ aggregates.	[115]
AD	Orange peel extract	200 mg/kg bw of extract for 6 weeks	Male albino rats	The extract was found to protect against AlCl_3_-induced neuronal damage by decreasing the activity of AChE, Aβ42 protein level, TBARS, and No level.	[116]
AD	Prickly pear	100 mg/kg bw of extract of pulp and peel	Rats	Attenuated AlCl_3_-induced learning and memory impairment. Significantly reduced the higher brain levels of proinflammatory cytokines	[117]
AD	Sweet pepper	20 µL of extract	Antioxidant activity and inhibition of key enzymes	The strongest antioxidant, anti-BChE, and anti-BACE1 activities were found in green sweet peppers. The highest AChE inhibition levels were found in yellow sweet pepper extract.	[101]
AD	*Solanum lyratum*	12.5, 25, 50 μM of solanoids F–I for 1 h	SH-SY5Y cells	Solanoids F–I exhibited neuroprotective effects against H_2_O_2_-induced oxidative damage of human SH-SY5Y cells.	[118]
AD	*Syagrus romanzoffiana* fruit and leaves	50 and 100 mg/kg bw of extract	AChE activity assays	Caused a decline in AChE activity and enhanced the histopathological changes in the cerebral cortex and cerebellum of the rat model of AlCl_3_-induced AD	[119]
AD	Virgin olive oil	50 mL of virgin olive oil daily for 12 months	Management of mild cognitive impairment patients’ clinical trial	AD-related biomarkers (p-tau, Aβ1–42, Aβ1–42/Aβ-40 ratio) returned to normal levels after administration of virgin olive oil	[103]
PD	Beans *(Vicia faba)*	600 mg/kg	Male Swiss albino mice	Antioxidant, anti-inflammatory, and neuroprotective effects	[120]
PD	*Carthamus tinctorius*	25, 50, and 100 mg/Kg of extract	C57BL/6 mice	Exerted neuroprotective effects on 6-OHDA-induced dyskinesia and dopaminergic neuron degeneration in PD mice; reduced the secretion of inflammatory factors via the attenuation of microglial NLRP3 inflammasome activation	[121]
PD	*Citrus trifoliata*	50 and 100 mg/kg	Manganese animal	Reduction of the striatal myeloperoxidase activity; renewal of dopamine, GABA and AChE; amelioration of neuronal apoptosis, microgliosis, and peri-neuronal vacuolation	[122]
PD	*Crocus Saitva*	50 mg/kg of *Crocus sativus* hydroethanolic extract	Meriones	Prevention of the development of PD resulting from lead (Pb)-induced nervous system damage, increasing TH levels in several brain areas including the substantia nigra compacta, locus coeruleus, dorsal striatum, and medial forebrain bundle.	[123]
PD	*Curcuma Longa*	0.001–0.4 mg/mL	SH-SY5Y human neuroblastoma cells	Amelioration of salsolinol-induced toxicity, reduction of mitochondria-derived ROS, and downregulation of caspase-3 activity.	[124]
PD	Florida beans (*mucuna pruriens)*	12.5–17.5 mg/kg	Swiss Albino mice	Improvement of motor response; reduction of dyskinesia.	[125]
PD	*Gastrodia elata*	2.5–40 μM of 20C (polyphenols)	PC12 cells	Amelioration of mitochondrial dysfunction; alleviation of PD by inhibition of α-Syn aggregation and maturation; maintaining the homeostasis of mitochondrial dynamics.	[126]
PD	*Ginkgo Biloba*	30–1500 μg/mL (leaf extract) in vitro; 50 mg/kg in vivo	Male C57BL/6 mice and SH-SY5Y human neuroblastoma cells	Protection of dopaminergic neurons against 6-OHDA and MPTP/MPP^+^-induced neurotoxicity	[127]
PD	*Ginseng*	2.5–40 mg/kg	Male Wistar rats	Attenuation of damage caused by toxicants in the nigra and the striatum by increased number of TH-positive cells; improved motor function.	[127]
PD	*Nicotiana tabacum*	200 μg/mL of extract	Mouse embryonic fibroblasts	Nicotine suppresses SIRT6 which confers resistance to neuron and cell death	[128]
PD	*Paeonia lactiflora*	1, 5, 10, 50, and 100 μM/L	Isolated primary neurons from pregnant female C57BL/6 (in vivo)	Neuroprotective effects against dopaminergic neuron degeneration, MPP^+^-induced ferroptosis via de Akt/Nrf2/GPC4 signaling pathway and, cerebral ischemia reperfusion-induced neuroinflammation and oxidative stress via Akt/Nrf2 activation.	[129]
PD	*Passiflora incarnata*	150 and 300 mg/kg (BEPIF: n-butanol extract of *Passiflora incarnata* flower)	Swiss albino mice and Sprague Dawley rats (in vivo)	Antioxidant activity that led to significant DPPH scavenging and H_2_O_2_ scavenging ability; reduced haloperidol-induced catalepsy and number of jaw movements induced by tacrine (an animal model of Parkinson tremors); protective effect in PD.	[7]
PD	*Polygonum Cuspidatum*	20–80 mg/kg of extract	Kun Ming mice	Restores MPTP-induced motor behavioral deficits; improves exercise endurance and circadian activity of MPTP-exposed mice; protects against MPTP-induced loss of dopaminergic nigrostriatal system; inhibits neuronal apoptosis.	[130]
PD	Seaweed *Bifurcaria bifurcata*	100 µg/mL	SH-SY5Y human neuroblastoma cells	Neuroprotective effects were mediated by the mitigation of ROS generation and mitochondrial dysfunctions, together with the reduction of Caspase-3 activity.	[131]
PD	*Scutellaria baicalensis*	5 mg/kg/per day of *Scutellaria baicalensis* stem-leaf total flavonoid	C57BL/6J male mice	Reduced damage to the dopaminergic neurons; inhibition of oxidation; alleviation in the damage of oxygen free radicals to dopaminergic neurons.	[132]
PD	*Scutellaria pinnatifida*	0.2, 0.3, 0.5, and 0.8 mM of neobaicalein	SH-SY5Y human neuroblastoma cells	Neobaicalein acted against oxidative stress, inflammation, and neurotoxins.	[40]
PD	St John’s Wort *(Hypericum Perforatum)*	1 mL; 300 mg/kg	C57BL/6 male mice	Reduced oxidative stress and improvement in ultrastructural changes in brain tissue of PD model; antioxidant and anti-inflammatory properties; neuroprotection against MPTP-induced PD model	[133]
PD	Tomatoes, grapefruits	25, 50, and 100 mg/kg b. wt., p.o	C57BL/6 male mice	Neuroprotection against MPTP-induced neurodegeneration in C57BL/6J mice; reduction of oxidative stress; neuroprotection by reducing neuroinflammation and improvement of motor function in MPTP-intoxicated mice	[134]
PD	Thunder God Vine *(Tripterygium wilfordii)*	0.1–3 µM celastrol	SH-SY5Y human neuroblastoma cells	Celastrol provided neuroprotection in PD by activating mitophagy to degrade damaged mitochondria and further inhibited dopaminergic neuronal apoptosis.	[135]

Abbreviations: AD—Alzheimer’s disease; AChE—acetylcholinesterase; AlCl_3_—aluminum chloride; Aβ42—amyloid beta; BACE1—β-site amyloid precursor protein cleaving enzyme 1; BChE—butyrylcholinesterase; bw—body weight; GABA—γ-amino butyric acid; TBARS—thiobarbituric acid-reactive substances.

## Data Availability

No new data were created or analyzed in this study. Data sharing is not applicable to this article.
